# Trans-Species Polymorphism in Immune Genes: General Pattern or MHC-Restricted Phenomenon?

**DOI:** 10.1155/2015/838035

**Published:** 2015-05-24

**Authors:** Martin Těšický, Michal Vinkler

**Affiliations:** Charles University in Prague, Faculty of Science, Department of Zoology, Viničná 7, 128 44 Praha, Czech Republic

## Abstract

Immunity exhibits extraordinarily high levels of variation. Evolution of the immune system in response to host-pathogen interactions in particular ecological contexts appears to be frequently associated with diversifying selection increasing the genetic variability. Many studies have documented that immunologically relevant polymorphism observed today may be tens of millions years old and may predate the emergence of present species. This pattern can be explained by the concept of trans-species polymorphism (TSP) predicting the maintenance and sharing of favourable functionally important alleles of immune-related genes between species due to ongoing balancing selection. Despite the generality of this concept explaining the long-lasting adaptive variation inherited from ancestors, current research in TSP has vastly focused only on major histocompatibility complex (MHC). In this review we summarise the evidence available on TSP in human and animal immune genes to reveal that TSP is not a MHC-specific evolutionary pattern. Further research should clearly pay more attention to the investigation of TSP in innate immune genes and especially pattern recognition receptors which are promising candidates for this type of evolution. More effort should also be made to distinguish TSP from convergent evolution and adaptive introgression. Identification of balanced TSP variants may represent an accurate approach in evolutionary medicine to recognise disease-resistance alleles.

## 1. Introduction

Immune function is highly heritable [1–4], governed from a large proportion by combination of alleles encoding functionally relevant immune-related molecules [5–7]. The alleles of immune genes coevolve in interaction with pathogens attacking the organism [8]. According to the Red Queen hypothesis pathogens form constant pressure on host population, selecting in many cases on variability within immune genes [9]. Genetic variability underlying the selected heterogeneity in the immune function is observable in the host as allelic polymorphism, that is, a long-lasting occurrence of two or more genotypes in a population in frequencies that cannot be attributed to a recurrent mutation [10]. Long-lasting polymorphism may be maintained in the human and animal host populations by balancing selection [11–13]. Intriguingly, this polymorphism maintained by selection may be shared across species and even between higher evolutionary lineages such as genera or rarely families [14–18]. This sharing of immunologically important genetic variation may have then profound effects on the interspecific similarity of naturally occurring ranges of immune responsiveness upon specific antigen stimulation.

**Figure 1:**
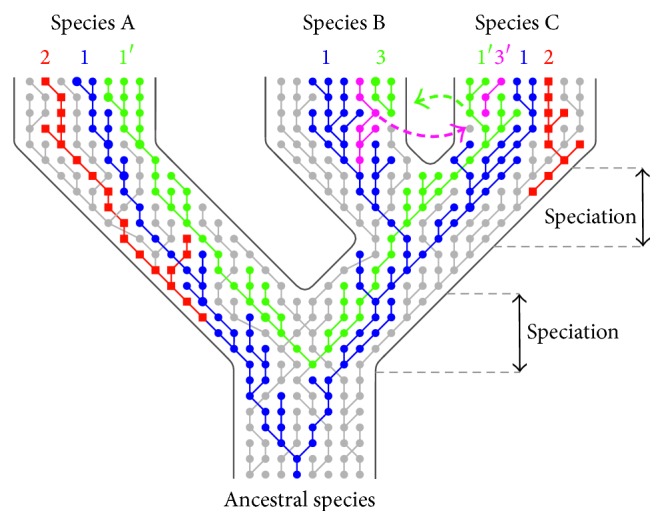
Mechanisms explaining polymorphism shared between taxa (based on [19, 112]). The three proposed mechanisms are depicted in alleles' genealogy: (1) trans-species polymorphism, TSP (incomplete lineage sorting; allelic lineages predate speciation and are passed to descendent species), (2) convergence (allelic lineages evolve similar features independently in separate lineages), and (3) introgression (allelic lineages are horizontally transferred either from recipient species to donor species or in both directions). Each row depicts a gene pool of one generation, each circle/square an allele of specific features. Different colours highlight individual allelic lineages, where interconnecting lines mark antecedent-descendent relationships. Green and purple dashed arrows represent directions of introgression.

Trans-species polymorphism (TSP) refers to the occurrence of identical or similar alleles in related species, excluding instances where the similarity arose by convergence or introgression [19, 20]. By definition, TSP alleles in related species are more similar in their sequences than are the alleles within individual species. TSP arises from the passage of alleles from ancestral species to descendant species by incomplete lineage sorting [19–21] (see also in Figure 1). Generally, we distinguish two forms of TSP, neutral TSP and balanced TSP. Neutral (transient) TSP is frequent in closely related newly diverged species and gradually disappears [19]. Thus, neutral TSP has a tendency to be widespread across loci only in a short window of time after the speciation event [22, 23]. In contrast, balanced TSP is functionally much more important [20]. This type of TSP results from balancing selection, that is, selection for variability maintenance. Balanced TSP is typically long-lasting and may be maintained in immune genes for millions or even tens of millions of years [24–26]. Identification of balanced TSP variants is, therefore, a powerful approach to identify naturally occurring resistance alleles with application potential in human medicine as well as in animal breeding and nature conservation.

The TSP concept was proposed three decades ago by Klein [27] who supported its existence by comparative evidence in major histocompatibility complex (MHC, H2 antigen) in mice [28]. Until present, MHC alleles from many mutually related species were sequenced and the TSP phenomenon was reported in numbers of studies in all sorts of taxa (see Supplement 1 in Supplementary Material available online at http://dx.doi.org/10.1155/2015/838035). Surprisingly, our knowledge on TSP in other immune gene classes is only limited. Is TSP unique to MHC or does it represent a general evolutionary pattern masked by little endeavour paid to its investigation outside the MHC family? In the present review we compile present evidence on TSP in human and animal immune genes and outline main directions for further evolutionary immunogenetic research.

## 2. Evolutionary Mechanisms Maintaining Balanced TSP in Immune Genes

Long-lasting TSP in immune genes is dependent on balancing selection. This type of selection maintains genetic variation in populations for extensive periods of time based on three possible mechanisms: (1) heterozygote advantage [29, 30], (2) negative frequency-dependent selection [31, 32], and (3) spatio-temporally fluctuating selection [33, 34]. Heterozygote advantage (also termed overdominance) arises when individuals heterozygous in a particular gene are able to resist the pathogen infection better than both homozygotes. In this case polymorphism is maintained by selection for heterozygosity. Overdominance is well described for MHC genes in a number of species where the benefits of heterozygosity in certain loci depend on the degree of overlap in binding specificity of individual alleles [30, 35]. Negative frequency-dependent selection, in contrast, represents a mechanism where only particular genotypes provide resistance advantage in a given time. This advantage is, nevertheless, negatively linked to the allele frequencies in a population [31]. It has been repeatedly shown that pathogens tend to infect and adapt to the most common genotypes of the host in a population, leaving out rare genotypes [36]. Rare alleles are, therefore, favoured and increase in frequency until they reach a specific equilibrium beyond which they start to be selected against. Frequencies of the alleles thus oscillate in time and balanced polymorphism is maintained [12, 30]. Empiric supports come from associations of MHC alleles of susceptibility to diseases [30, 35, 36]. Finally, fluctuating selection is based on variation in selective pressures in space and time. Most pathogens are present in a limited area of distribution and their abundance change in time (spatio-temporal fluctuations). This creates distinct and changing selective pressures on different host populations that may be, nonetheless, linked by migration [33, 34]. In contrast to the negative frequency-dependent selection, in fluctuating selection the fitness value changes as a function of particular pathogen abundance and not as a function of the allele frequency itself [33]. Although there is still serious lack in empirical evidence supporting the existence of fluctuating selection, theoretical approaches suggest that this mechanism is admissible, for instance, for the maintenance of the MHC polymorphism [12]. The three mechanisms of balancing selection are not mutually exclusive and in all perceivable combinations all may be involved in the maintenance of the balanced TSP.

## 3. TSP in Major Histocompatibility Genes (MHC)

**Figure 2:**
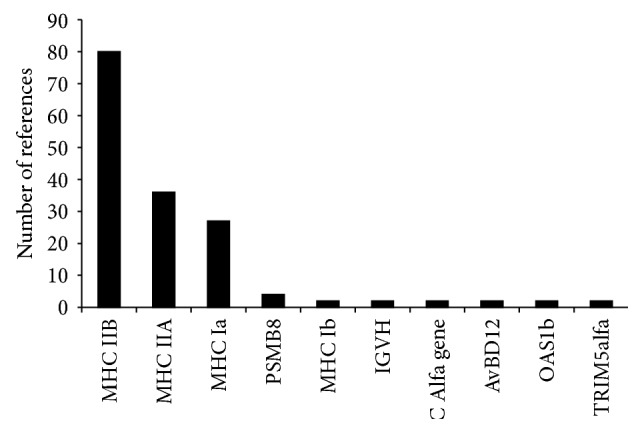
Number of published research articles dealing with TSP in vertebrate immune genes available on Web of Science, final update 19 March 2015. For details see Supplement 1.

MHC groups several extremely polymorphic and dynamically evolving members of the immunoglobulin superfamily playing a crucial role in the adaptive immune defence against pathogens in jawed vertebrates [29, 37]. MHC genes encode cytoplasm-membrane-bound glycoproteins which present endogenous and exogenous oligopeptides to T cells [38]. Although the strength of the association between MHC polymorphism and resistance to infectious diseases varies between species and populations [39, 40], the capability of certain alleles to bind certain pathogen-derived peptides is undoubtedly essential for individual survival [30]. The type of the selection responsible for the variability maintenance linked to disease resistance may, however, not only be natural selection but also sexual selection [31, 41], since the two mechanisms are not mutually exclusive and both can contribute to balanced parasite-mediated polymorphism. The MHC gene family can be generally divided into two classes: MHC class I and MHC class II [39]. Owing to their importance in the immune response and a high variability on both interspecific and intraspecific levels, traditionally, most studies dealing with TSP have focused on MHC class I and MHC class II genes and their peptide binding regions (PBRs) in particular (summarized in Table 1 and Figure 2).

MHC class I proteins consist of a transmembrane *α*-chain composed of *α*_1_, *α*_2_, *α*_3_ domains and *β*_2_-microglobulin [29, 30, 38]. PBR is coded by exon 2 (*α*_1_-domain) and exon 3 (*α*_2_-domain) and binds shorter oligopeptide fragments (approximately 8–11 amino acids in length) originating from an intracellular pathogen or endogenous peptides. Complexes of MHC class I molecules with their peptides are recognized by T-cell receptors (TCRs) of CD8^+^ T cells. MHC class II molecules, in contrast, consist of two noncovalently associated chains: *α* chains (*α*_1_, *α*_2_) and *β* chains (*β*_1_, *β*_2_), encoded by two different genes termed MHC IIA and MHC IIB [30, 32]. The MHC class II PBR is formed by N-terminal domains of these molecules—*α*_1_ (exon 2) and *β*_1_ (exon 2). Opened binding groove of MHC class II allows binding of longer peptides (approximately 11–17 amino acids in length) originating from extracellular pathogens or intracellular pathogens inhabiting vesicular systems [29, 30]. Complexes of MHC class II molecules with their peptides are recognized by CD4^+^ T cells [29, 38]. However, cross presentation of antigenic peptides enables the exposure of some MHC-class-II-type exogenous peptides on the binding surface of MHC class I molecules, and vice versa [38, 42].

In MHC genes TSP encompasses mainly variable exons encoding PBRs (in MHC class I exons 2 and 3, in MHC class II exon 2 of both MHC IIA and MHC IIB genes) that have been the targets of strong diversifying (increasing allele numbers) and positive (adapting the alleles to pathogenic peptides) selection followed by balancing selection modifying allele frequencies [19, 43]. There are highly variable and divergent alleles which can persist as identical or nearly identical alleles within millions of years [16] and as allelic lineages up to tens of millions of years [19]. In contrast to PBR, non-PBR MHC domains are usually evolutionary conserved, often under purifying selection without or with minimal TSP [26, 44–46]. Although evidence for TSP has been gathered from several MHC loci in mammals, birds, reptiles, amphibians, and fish indicating that TSP is a general phenomenon in MHC, the vast majority of studies focused on genotyping only the most variable exon 2 of the MHC IIB genes (Table 1 and Figure 2). In MHC IIA loci the lower number of studies reporting TSP may be explained by the general belief of their more conserved nature when compared to MHC IIB genes [24, 47, 48]. Furthermore, despite the broad range of taxa investigated, most evidence on TSP is limited to mammals (especially primates, rodents, and even-toed ungulates; see Supplement 1).

Time persistence of allelic lineages in mammalian classical MHC class I genes is assumed to be generally shorter than in classical MHC class II genes [19, 49, 50]. For instance, in primates MHC class I allelic lineages HLA-A, -B, -C are probably younger than 22 million years [19, 49]. In contrast, some allelic lineages in primate MHC class II appear to be older than 30 million years [51]. In Lemuriformes identical MHC class II alleles of DRB exon 2 persist for more than 37–42 million years [17]. Similarly, in DQA genes in rodents, the oldest alleles are maintained for at least 48 million years [52]. Interestingly, TSP is maintained in fish, amphibians, and reptiles on much longer timescales than in mammals [45, 53–55]. For instance, the oldest allelic lineages of exon 2 MHC Ia that are shared between Salmoniformes and Cypriniformes precede the taxa divergence and persist for more than 145 million years [56]. The oldest reported TSP then concerns antigen presenting MHC class I within Acipenseriformes, with time persistence of 187 million years [57].

## 4. Trans-Species Polymorphism in Other Immune Genes

### 4.1. Non-MHC Immunoglobulins

TSP has also been described in members of the immunoglobulin superfamily other than MHC. The existence of high genetic variability and TSP in heavy chains of immunoglobulins in rabbits and hares has been predicted by serological cross reactivity among serotypes as early as in 1980s [58]. Heavy chains of immunoglobulins are encoded by the immunoglobulin variable region heavy chain (IgVH) genes [59]. Variability in these genes may affect the binding specificity of antigen binding sites of antibodies. Apart from IgVH genes directly involved in VDJ arrangements, other IgVH genes including pseudogenes can serve as a source of variation for gene conversion [60]. It has been shown that the IgVH TSP in the Leporid lineage concerns the VH1/VHa gene and predates the rabbit and hare divergence and persists for at least 16–24 million years, perhaps as long as for 50 million years [58, 60, 61]. Another example of TSP in the immunoglobulin superfamily has been reported among eight macaque species in exon 2 of the C*α* constant region of IgA heavy chain [62, 63]. Although previously considered as conservative, high interspecific as well as intraspecific variability has been discovered in primates [62–64]. Exon 2 encodes the hinge region which lies at the base of the heavy chain regions of immunoglobulin [62, 63]. Variability in the hinge region associated with variation in flexibility of the molecule might affect the spectrum of antigens recognised by the antigen binding site of the IgA antibody [62] or allow avoidance of the attacks of bacterial proteases in this region [62, 65].

### 4.2. PSMB8

PSMB8 (proteasome subunit *β*-type 8 gene, LMP7) encodes immunoproteasomal catalytic subunit of *β*-ring which is involved in the cleavage of peptides processed for presentation on MHC class I molecules [66]. This gene is located in MHC class I gene cluster, and together with another proteasomal gene PSMB9 its expression is interferon-induced. Polymorphism in this gene involves amino acid position 31 that affects the catalytic function of the subunit. Two functionally distinct allelic lineages differing at this position have been distinguished in various vertebrate lineages [67]: (1) A-type PSMB8 having Ala/Val at the position 31 with a larger and opened S-pocket allowing the cleavage of aromatic amino acids with the chymotrypsin activity and (2) F-type PSMB8 having Phe/Tyr at the position of 31 with a narrower S-pocket suitable for elastase cleavage of small hydrophobic amino acids [67]. This type of TSP has been reported in PSMB8 in bony fish of the* Oryzias* genus [68], where two highly diverged allelic lineages differing in the sequence encoding the S-pocket (called here PSMB8N and PSMB8d) persist for at least 30–60 million years. A similar pattern has also been reported among clawed frog (*Xenopus*) species [69] suggesting the existence of similar independent TSP allelic lineages in amphibians for 80 million years. Intriguingly, allelic dichotomy in PSMB8 gene between Cypriniformes and Salmoniformes (PSMB8F and PSMBA) suggests extremely long-term transorder polymorphism maintained for more than 300 million years [67]. To conclude, these examples demonstrate long-term TSP in lower taxonomic units (Oryzias, Xenopus and between Salmoniformes and Cypriniformes) in genetic traits that most probably converged between major vertebrate lineages [67, 70].

### 4.3. Host Defence Peptides

Host defence peptides (HDPs, also known as antimicrobial proteins) are small, diverse, and evolutionary conserved effector molecules involved mainly in pathogen killing [71, 72], but also in immunomodulation, wound healing, cell development, and so forth [73, 74]. There are many types of HDPs in all sorts of organisms [75], and HDP intraspecific sequence variability maintained by balancing selection has been reported [76, 77]. In HDPs, TSP has been documented so far only in avian *β*-defensin AvBD12 gene [78]. *β*-defensins are amphipathic cationic cysteine-rich peptides [72]. In humans, intraspecific polymorphism in *β*-defensin genes affects susceptibility to pathogens, such as HIV [79]. In AvBD12 gene TSP was described in exon 3 in two passerine species: blue tits and great tits [78]. Given the limited sampling effort more widespread occurrence of TSP in HDPs cannot be excluded.

### 4.4. TRIM5*α*

TRIM5*α* (tripartite motif protein 5, *α*-isoform) is the longest isoform of a viral restriction factor which interacts with viral capsid proteins in cytosol during retrovirus infection and thus prevents reverse transcription [80, 81]. Similarly to MHC genes, TRIM genes are highly polymorphic and their evolution has been driven by gene loss, pseudogenization, duplication, and punctuated positive selection [80, 82]. TRIM5*α* consists of C-terminal SPRY/B30.2 domain, RING domain, and B-box2a and coiled-coil (CC). TSP is documented among macaques in the SPRY/B30.2 domain and among biting midges (*Ceratopogonidae*) in the CC domain [82]. The variability in SPRY/B30.2 domain determinates restriction specificity [80], while the functional significance of the polymorphism in CC domain still remains unresolved [80, 82]. In addition to the TSP in coding region of the gene, TSP has also been detected in intron 1, apparently maintained between humans and chimpanzees by balancing selection for 4–7 million years [83]. This TSP may affect transcriptional activity of the gene.

### 4.5. Oligoadenylate Synthetase

Oligoadenylate synthetases (OASs) are interferon-inducible enzymes with pleiotropic functions involved in the organism protection against retroviral infections [84]. After activation by interferons the RNA-dependent 2′,5′-OAS synthesizes 2′,5′-oligoadenylates from adenosine triphosphate, which activates latent endoribonuclease RNase L leading to degradation of dsRNA and inhibition of viral replication [84]. In mice, the variability in gene OAS1b has functional consequences on variation in resistance against flavivirus infections, for example, to West Nile virus [85, 86]. Species of Palearctic mice share two deeply diverged allelic lineages of OAS1b predating the split of house mouse (*Mus musculus*) and servant mouse (*M. famulus*) 2.8 million years ago. Both groups of allelic lineages provide resistance to flavivirus infections; however, the lineage of “major resistance alleles” protects against a broader spectrum of flavivirus genotypes compared to “minors resistance alleles” [87]. This TSP appears to involve only the C-terminal domain of OAS1b which is responsible for the enzyme tetramerization and protein-protein binding [87]. In contrast, the OAS1 TSP known in humans, chimpanzees, and gorillas concerns the N-terminal RNA-binding region [88].

## 5. Future Directions

TSP is a crucial evolutionary mechanism responsible for sharing adaptive genetic variation across taxa. Although presently most studies dealing with TSP have concentrated only on the MHC loci (*MHC I* and* MHC II* and their peptide binding regions, in particular), the few examples described in other immune genes suggest that TSP is a common and general evolutionary phenomenon. Indeed, besides immune genes, the TSP has been documented, for example, in self-incompatibility loci preventing self-fertilization in Angiosperms [89–91], in mating loci in fungi [92–94], in ABO blood system in primates [95–97], or in complementary sex determiner gene in Hymenoptera [98, 99]. Investigation of TSP predominantly in association with MHC thus appears to be a historically given stereotype which might have slowed down the rate of similar investigation in other families of immune genes. It has been recently proposed for humans-chimpanzees that TSP is especially common in membrane glycoproteins [100]. As shown in the present review, TSP may be present in genes encoding effector molecules as well as immune receptors. The products of both gene types physically interact with pathogen molecules and are, therefore, more likely than others to evolve under balancing selection.

Further research should clearly focus more intensively on investigation of TSP patterns in innate immune genes. It has been proposed recently that at least approximately half of the genetic variability for resistance to infection is attributable to non-MHC genes [101]. We suggest that besides antimicrobial peptides, oligoadenylate synthetases, or viral restriction factors where the first evidence of TSP has been reported, further effort should be made to reveal TSP in pattern recognition receptors (PRRs). PRRs are innate immunity receptors that recognize pathogen-associated molecular patterns (PAMPs) that serve as danger signals [102]. Considering the direct physical association between PRRs and PAMPs in triggering the immune response [103], in concordance with the Red Queen hypothesis we may predict strong evolutionary pressures maintaining balanced frequencies of PRR alleles. There are many families of PRRs, toll-like receptors (TLRs), C-type lectin receptor (CLRs), RIG-like receptors (RLSs, retinoic acid-inducible gene-I-like receptors), NOD-like receptors (NLRs, nucleotide-binding oligomerization domain receptors), and others [102, 104]. Despite the fact that PRRs are evolutionary relatively conserved, considerable nonsynonymous polymorphism with predicted functional significance has been recently documented in the binding sites of these receptors both on interspecific and intraspecific levels [105–109]. Recent comparison of human populations also revealed that balancing selection is the main force shaping the evolution of many innate immunity genes [110]. PRRs, hence, appear as suitable candidate genes for TSP investigation.

Although commonly assessed in gene coding regions, TSP may also be maintained in other functionally important parts of the genome, such as, for example, the promoter regulatory sequences of individual genes. It has been reported in primates that TSP may preferentially involve transcription factor binding sites regulating gene expression [100, 111]. Our knowledge on TSP in noncoding regions is currently insufficient and further research is needed to show how widespread this type of TSP is.

When studying patterns of polymorphism on interspecific level, some caution is also needed before TSP is assigned and reported. Several evolutionary mechanisms other than balanced TSP were also proposed to explain the existence of shared polymorphism in several related taxa. These are, namely, (1) incomplete lineage sorting by chance (sometimes described as neutral or transient TSP as described in the introduction), (2) convergent evolution, and (3) genetic introgression. Distinguishing these mechanisms from TSP is one of the greatest challenges in the present TSP research [112, 113] (see also Figure 1). In contrast to balanced TSP and incomplete lineage sorting by chance (which is a state expected mainly in newly diverged species), convergent evolution is a process whereby organisms (related or not) independently evolve similar traits as a result of adaptation to similar environments or ecological niches [20, 32]. Although presumably common in immune genes [32, 51, 114], convergence has been shown difficult to detect. Convergent evolution usually operates in short functionally important motifs and may be, therefore, distinguished from TSP by specific coding resemblance only in these key regions [32]. Comparison of phylogenetic trees constructed based on regions with a distinct function (e.g., peptide-binding region, PBR, sequence and non-PBR sequence of MHC) may be used to differentiate between TSP and convergence [26, 51, 114]. Also the third mechanism that may be mistaken with TSP, that is, hybridization with subsequent adaptive introgression, may be far more common than generally assumed [112]. Introgression occurs mainly in evolutionary young, radiated, or closely related species with incomplete reproductive isolation mechanisms, which further complicates its differentiation from TSP. Mixing alleles of the both trans-specific and hybrid origin (that are barely distinguishable) have been reported in adaptive radiated species, in Darwin finches [115, 116] or cichlid fish of* Haplochromis* species flock of East African lakes [23]. Furthermore, hybridization can also occur in relatively diverged taxa. Almost one-tenth of bird species may hybridize [117]. There is still lack of evidence to show how common is the hybridization-linked adaptive introgression in immune genes (but see, e.g., [14, 112, 113, 118–120]). One of the possible ways to distinguish between adaptive introgression and TSP is to compare the relative size of the haplotype blocks of the shared polymorphism. In case of TSP we expect these blocks to be smaller than in case of the gene introgression [113, 118, 119]. Combining large data sets with novel genomic approaches such as the highly dense SNP chips or next-generation sequencing may be useful in doing so. Not only basic evolutionary research but also human and veterinary medical research may benefit from precise identification of TSP. In addition to the clear potential of TSP to identify functionally important variation in human and animal genomes (that may be associated with disease susceptibility or resistance), TSP alleles may also be linked to deleterious mutations accumulated in the neighbourhood of the sites maintained by the balancing selection [121]. Identification of beneficial and deleterious variability in genomes is essential precondition allowing complex personalised medicine in the future.

## 6. Conclusion

Taken all together, despite its importance and general applicability, in recent research the TSP concept is being commonly associated only with MHC trans-specific genetic variation. This research stereotype is unfortunate, since lacking evidence on TSP in other types of immune genes precludes our understanding of the mechanisms shaping the present genetic variability maintained by host-pathogen coevolution. Besides focusing on TSP in innate immunity genes (and mainly PRRs), more effort should also be made to distinguish true TSP from TSP-like patterns. Although originally a question of an academic interest, TSP investigation may bring practically relevant results. Identification of TSP variants is a powerful approach to identification of naturally occurring resistance alleles with application potential in human and veterinary medicine, animal breeding, and nature conservation. Recent advance of genomics in immunology allows us systematic research of TSP in all sorts of immune genes and perceiving the TSP concept as a MHC-linked mechanism would jeopardise the interpretational potential of the current immunogenetic research.

## Supplementary Material

Supplement 1 lists published research articles dealing with TSP in vertebrate immune genes available on Web of Science [final update 19th March 2015]. Articles dealing with TSP in blood group systems and serological allomorphs were not included into this survey.

## Figures and Tables

**Table 1:** Number of published research articles dealing with TSP in vertebrate immune genes available on Web of Science, final update 19 March 2015. For details see Supplement 1.

Gene group	Gene	Number of references
Major histocompatibility complex (MHC)	Classical MHC I: HLA-A, -B, -C, MHC Ia undifferentiated	27
	Non-classical MHC I: HLA-E, -G	2
	MHC IIA: DPA, DRA, DQA, DAA, MHC IIA undifferentiated	36
	MHC IIB: DPB, DRB, DQB, DAB, DRB-like, MHC IIB undifferentiated	80

Non-MHC immunoglobulins	PSMB8 (LMP7)	4
	IGVH	2
	C Alfa gene	2

Host defence peptides	AvBD12	2

2-5A synthetase family	OAS1b	2

Tripartite motif protein family	TRIM5alfa	2
