# Supplementary material for: Trans-Species Polymorphism in Immune Genes: General Pattern or MHC-Restricted Phenomenon?
Source: J Immunol Res. 2015 May 24;2015:838035. doi: 10.1155/2015/838035 (PMC4458282; doi:10.1155/2015/838035)
Supplement: Supplementary file 1 — Supplement 1 lists published research articles dealing with TSP in vertebrate immune genes available on Web of Science [final update 19th March 2015]. Articles dealing with TSP in blood group systems and serological allomorphs were not included into this survey. [file 838035.f1.pdf]

Supplement 1: List of published research articles dealing with TSP in vertebrate immune genes available on Web of Science [final update 19th March 2015]. Given by the variability in terminology adopted by different authors the articles have been systematically searched by the following formula: Topic: “Trans-species polymorphism” OR “Tran-species polymorphism” OR “Transspecies polymorphism” OR “Transpecies polymorphism ”OR” Trans-species evolution” OR ”Tran-species evolution” OR ”Transspecies evolution” OR ”Transpecies evolution” OR “Trans-specific polymorphism” OR “Tran-specific polymorphism” OR “Transspecific polymorphism” OR “Transpecific polymorphism” OR ”Trans-specific evolution” OR ”Tran-specific evolution” OR ”Transspecific evolution” OR ”Transpecific evolution” OR “shared polymorphism”. Articles dealing with TSP in blood group systems and serological allomorphs were not included into this survey. MHC – Major histocompatibility complex, Ig – Immunoglobulins, PSMB8 – Proteasome subunit beta-type 8 gene (syn. LMP7), IgVH - Immunoglobulin heavy chain gene, HDP – Host-defence peptides, AvBD12 – Avian beta beta-defensin 12, OAS – Oligoadenylat synthethase, TRIM – Tripartite motif protein, CDS – Coding DNA sequence, Ex. – exon, Int. – Intron, undif. – undifferentiated, Ref. – Reference. Gene subregions in round brackets indicate TSP detected in whole fragment, in contrast to gene segments connected with “and” indicating TSP reported independently for separate regions. Taxon includes only the taxonomy units with TSP detected.

**Gene group**	**Gene name**	**Gene function**	**Gene subregion**	**Taxon**	**Taxon (scientific)**	**Ref.**
MHC	MHC I-A	antigen presentation	CDS	Mammals: cercopithecine monkeys	Cercopithecoidea	[1]
MHC	MHC I-A	antigen presentation	ex. 2 and ex. 3	Mammals: primates	Primates	[2]
MHC	MHC I-A	antigen presentation	CDS	Mammals: human and chimpanze	*Pan* and *Homo*	[3]
MHC	MHC I-B	antigen presentation	CDS	Mammals: cercopithecine monkeys	Cercopithecoidea	[1]
MHC	MHC I-B	antigen presentation	ex. 2 and ex. 3	Mammals: primates	Primates	[2]
MHC	MHC I-B	antigen presentation	CDS	Mammals: human and chimpanze	*Pan* and *Homo*	[3]
MHC	MHC I-C	antigen presentation	ex. 2 and ex. 3	Mammals: primates	Primates	[2]
MHC	MHC Ia (undif.)	antigen presentation	ex. 2 and ex. 3	Mammals: night monkeys	*Asio*	[4]
MHC	MHC Ia (undif.)	antigen presentation	(ex. 2, 3)	Mammals: bisons	*Bison bonasus* and *Bison bison*	[5]
MHC	MHC Ia (undif.)	antigen presentation	ex. 2	Mammals: cetaceans	Cetacea	[6]
MHC	MHC Ia (undif.)	antigen presentation	ex. 2	Mammals: carnivores	*Meles meles* and *Taxidoea taxus* and other Ursiadae,Caniadae, Phociane	[7]
MHC	MHC Ia (undif.)	antigen presentation	ex. 2	Mammals: felids	Felidae	[8]
MHC	MHC Ia (undif.)	antigen presentation	ex. 2	Mammals: bears	Ursidae	[9]
MHC	MHC Ia (undif.)	antigen presentation	ex. 3	Birds: songbirds	*Passer* and *Acrocephalus*	[10]
MHC	MHC Ia (undif.)	antigen presentation	ex. 3	Birds: songbirds	*Acrocephallus arundicaneus* and *Acrocephallus sechelensis*	[11]
MHC	MHC Ia (undif.)	antigen presentation	ex. 4	Reptiles: lizards	*Eremias*	[12]
MHC	MHC Ia (undif.)	antigen presentation	ex. 2 and ex. 3	Reptiles: iguanas	Iguanidae	[13]
MHC	MHC Ia (undif.)	antigen presentation	ex. 2	Reptiles: sea turtle and other reptiles	*Carreta caretta* with other reptiles	[14]
MHC	MHC Ia (undif.)	antigen presentation	ex. 2 ex. 3	Amphibians: shrub frogs	Rhapcoporiadae species and	[15]

Gene group	Gene name	Gene function	Gene subregion	Taxon	Taxon (scientific)	Ref.
					other frog families	
MHC	MHC Ia (undif.)	antigen presentation	ex. 2 and ex. 3	Amphibians: frogs	Hylidae, Centrolenidae, Ranidae	[16]
MHC	MHC Ia (undif.)	antigen presentation	ex. 2 and ex. 3 and ex. 4)	Amphibians: frogs	*Xenopus*	[17]
MHC	MHC Ia (undif.)	antigen presentation	ex. 2 and ex. 3	Fish: paddlefish and Chinese sturgeon	*Polyodon spathula* and *Acipenser sinensis*	[18]
MHC	MHC Ia (undif.)	antigen presentation	gene fragment	Fish : basses	*Morone*	[19]
MHC	MHC Ia (undif.)	antigen presentation	(ex. 2, 3)	Fish: salmonids	Salmonide	[20]
MHC	MHC Ia (undif.)	antigen presentation	ex. 2 and ex. 3 and ex. 4	Fish: bone fish	Salmoniformes and Cypriniformes	[21]
MHC	MHC Ia (undif.)	antigen presentation	ex. 2 and ex. 3	Fish: white fish with other salmonids	*Coregonus* with other salmonids	[22]
MHC	MHC Ia (undif.)	antigen presentation	ex. 2	Fish: salmonids	Salmonidae	[23]
MHC	MHC I-E	antigen presentation	ex. 2 and ex. 3	Mammals: cercopithecine monkeys	Cercopithecoidea	[24]
MHC	MHC I-G	antigen presentation	ex. 2	Mammals: old world monkeys	Pongiadae, Cercopitheciane	[25]
MHC	MHC IIA (DPA)	antigen presentation	ex. 2	Mammals: old world monkey	Catarrhini	[26]
MHC	MHC IIA (DRA)	antigen presentation	ex. 2	Mammals: primates	Primates	[27]
MHC	MHC IIA (DRA)	antigen presentation	ex. 2	Mammals: old world monkey	Catarrhini	[26]
MHC	MHC IIA (DRA)	antigen presentation	ex. 2	Mammals: blesmoles and mole rats	Bathyergidae	[28]
MHC	MHC IIA (DRA)	antigen presentation	ex. 2	Mammals: bovids	Bovinae	[29]
MHC	MHC IIA (DRA)	antigen presentation	ex. 2	Mammals: bovids	Boviane	[30]
MHC	MHC IIA (DRA)	antigen presentation	ex. 2	Mammals: cetaceans	Cetacea	[6]
MHC	MHC IIA (DRA)	antigen presentation	ex. 2	Mammals: horses	*Equus*	[31]
MHC	MHC IIA (DRA)	antigen presentation	ex. 2	Mammals: horses	*Equus*	[32]
MHC	MHC IIA (DRA)	antigen presentation	ex. 2	Mammals: horses	*Equus*	[33]
MHC	MHC IIA (DQA)	antigen presentation	ex. 2	Mammals: primates	Primates	[27]
MHC	MHC IIA (DQA)	antigen presentation	promotor region	Mammals: primates	Primates	[34]
MHC	MHC IIA (DQA)	antigen presentation	ex. 2	Mammals: rodents	Rodentia	[35]
MHC	MHC IIA (DQA)	antigen presentation	(int. 1, ex. 2)	Mammals: tuco-tucos	*Ctenomys*	[36]
MHC	MHC IIA (DQA)	antigen presentation	ex. 2	Mammals: hares and rabbits	*Lepus* and *Oryctolagus*	[37]
MHC	MHC IIA (DQA)	antigen presentation	ex. 2	Mammals: hares and rabbits	*Lepus* and *Oryctolagus*	[35]
MHC	MHC IIA (DQA)	antigen presentation	ex. 2	Mammals: hares and rabbits	Leporidae	[38]
MHC	MHC IIA (DQA)	antigen presentation	ex. 2	Mammals: voles	Arvicolinae	[39]
MHC	MHC IIA (DQA)	antigen presentation	ex. 2	Mammals: carnivores	*Meles meles* and *Taxidoea taxus* and with other carnivores	[40]
MHC	MHC IIA (DQA)	antigen presentation	ex. 2	Mammals: canids	Canidae	[31]
MHC	MHC IIA (DQA)	antigen presentation	ex. 2	Mammals: bears	Ursiadae	[9]
MHC	MHC IIA (DQA)	antigen presentation	ex. 2	Mammals: domestic pig and wild boar	*Sus scrofa* f. *domestica* and *Sus scrofa*	[41]
MHC	MHC IIA (DQA)	antigen presentation	ex. 2	Mammals: caprids and bovids	Caprinae and Bovidae	[42]

Gene group	Gene name	Gene function	Gene subregion	Taxon	Taxon (scientific)	Ref.
MHC	MHC IIA (DQA)	antigen presentation	ex. 2	Mammals: sheeps	*Ovies*	[43]
MHC	MHC IIA (DQA)	antigen presentation	ex. 2	Mammals: bovids	Bovidae	[44]
MHC	MHC IIA (DQA)	antigen presentation	ex. 2	Mammals: ruminants	Bovianae and Cervidae	[45]
MHC	MHC IIA (DQA)	antigen presentation	ex. 2	Mammals: cetaceans	Cetacea	[6]
MHC	MHC IIA (DQA)	antigen presentation	ex. 2	Mammals: horses	*Equus*	[32]
MHC	MHC IIA (DQA)	antigen presentation	ex. 2	Mammals: horses	*Equus*	[33]
MHC	MHC IIA (DQA)	antigen presentation	ex. 2 and int. 2	Mammals: elephants	*Elephas* and *Loxodonta*	[46]
MHC	MHC IIA (DAA)	antigen presentation	ex. 2	Fish: salmons	*Salmo*	[47]
MHC	MHC IIA (DAA)	antigen presentation	ex. 2	Fish: danio	*Danio*	[48]
MHC	MHC IIA (undif.)	antigen presentation	ex. 2	Mammals: mice	*Mus*	[49]
MHC	MHC IIA (undif.)	antigen presentation	(ex. 2, 3)	Reptiles: crocodiles	Crocodilia	[50]
MHC	MHC IIB (DPB)	antigen presentation	ex. 2	Mammals: macaques	*Macaca*	[51]
MHC	MHC IIB (DPB)	antigen presentation	ex. 2	Mammals: macaques	*Macaca*	[52]
MHC	MHC IIB (DRB)	antigen presentation	ex. 2	Mammals: old world monkey	Catarrhini	[53]
MHC	MHC IIB (DRB)	antigen presentation	ex. 2	Mammals: primates	*Rhinopithecus roxellana* and other primates	[54]
MHC	MHC IIB (DRB)	antigen presentation	ex. 2	Mammals: primates	Primates	[2]
MHC	MHC IIB (DRB)	antigen presentation	ex. 2	Mammals: old world monkey	Catarrhini	[55]
MHC	MHC IIB (DRB)	antigen presentation	ex. 2	Mammals: old world monkey	Catarrhini	[56]
MHC	MHC IIB (DRB)	antigen presentation	ex. 2	Mammals: primates	Primates	[57]
MHC	MHC IIB (DRB)	antigen presentation	ex. 2	Mammals: primates	Primates	[58]
MHC	MHC IIB (DRB)	antigen presentation	ex. 2	Mammals: old world monkey	Catarrhini	[59]
MHC	MHC IIB (DRB)	antigen presentation	ex. 2	Mammals: primates	Primates	[60]
MHC	MHC IIB (DRB)	antigen presentation	ex. 2	Mammals: hominoids	Hominoidea	[61]
MHC	MHC IIB (DRB)	antigen presentation	ex. 2	Mammals: old word monkeys	Catarrhini	[62]
MHC	MHC IIB (DRB)	antigen presentation	ex. 2	Mammals: hominoids	Hominoidea	[63]
MHC	MHC IIB (DRB)	antigen presentation	ex. 2	Mammals: hominoids	Hominoidea	[60]
MHC	MHC IIB (DRB)	antigen presentation	ex. 2	Mammals: lemurs	Lemuriformes	[64]
MHC	MHC IIB (DRB)	antigen presentation	ex. 2	Mammals: rodents	*Sylvaticus* with other Muridae	[65]
MHC	MHC IIB (DRB)	antigen presentation	ex. 2	Mammals: rodents	Rodentia	[66]
MHC	MHC IIB (DRB)	antigen presentation	(int. 1, ex. 2)	Mammals: tuco-tucos	*Ctenomys*	[36]
MHC	MHC IIB (DRB)	antigen presentation	ex. 2	Mammals: rodents	Rodentia	[67]
MHC	MHC IIB (DRB)	antigen presentation	ex. 2	Mammals: carnivores	Ursidae and Felidae	[68]
MHC	MHC IIB (DRB)	antigen presentation	ex. 2	Mammals: carnivores	Ursidae and Felidae	[69]
MHC	MHC IIB (DRB)	antigen presentation	ex. 2	Mammals: hyeanas	Hyaeninae	[70]
MHC	MHC IIB (DRB)	antigen presentation	ex. 2	Mammals: canids	Canidae	[71]

Gene group	Gene name	Gene function	Gene subregion	Taxon	Taxon (scientific)	Ref.
MHC	MHC IIB (DRB)	antigen presentation	ex. 2	Mammals: minks	Mustelinae	[72]
MHC	MHC IIB (DRB)	antigen presentation	ex. 2	Mammals: felids	Felidae	[73]
MHC	MHC IIB (DRB)	antigen presentation	ex. 2	Mammals: canids	Canidae	[31]
MHC	MHC IIB (DRB)	antigen presentation	ex. 2	Mammals: bears	Ursiadae	[9]
MHC	MHC IIB (DRB)	antigen presentation	ex. 2	Mammals: carnivores	*Meles meles* and *Taxidoea taxus* and with other carnivores	[40]
MHC	MHC IIB (DRB)	antigen presentation	ex. 2	Mammals: felids	Felidae	[8]
MHC	MHC IIB (DRB)	antigen presentation	ex. 2	Mammals: ungulates	Boviane, Caprinae, Cervidae, Alcelaphinae	[74]
MHC	MHC IIB (DRB)	antigen presentation	ex. 2	Mammals: ruminants	within Cervinae and beetween Cervinae and Caprinae	[75]
MHC	MHC IIB (DRB)	antigen presentation	ex. 2	Mammals: ruminants	Boviane	[30]
MHC	MHC IIB (DRB)	antigen presentation	ex. 2	Mammals: ruminants	Boviane	[76]
MHC	MHC IIB (DRB)	antigen presentation	ex. 2	Mammals: sheeps	*Ovies*	[77]
MHC	MHC IIB (DRB)	antigen presentation	ex. 2	Mammals: caprids	Caprinae	[78]
MHC	MHC IIB (DRB)	antigen presentation	ex. 2	Mammals: cervids	Cervidae	[79]
MHC	MHC IIB (DRB)	antigen presentation	ex. 2	Mammals: cervids	Cervidae	[80]
MHC	MHC IIB (DRB)	antigen presentation	ex. 2	Mammals: bovids	Bovinae	[81]
MHC	MHC IIB (DRB)	antigen presentation	ex. 2	Mammals: cervids	Cervidae	[82]
MHC	MHC IIB (DRB)	antigen presentation	ex. 2	Mammals: domestic pig and wild boar	*Sus scrofa* f. *domestica* and *Sus scrofa*	[41]
MHC	MHC IIB (DRB)	antigen presentation	ex. 2	Mammals: bats	within *Noctillo* and *Myotis*	[83]
MHC	MHC IIB (DRB)	antigen presentation	ex. 2	Birds: auclets	*Aethia*	[84]
MHC	MHC IIB (DQB)	antigen presentation	ex. 2	Mammals: primates	Primates	[85]
MHC	MHC IIB (DQB)	antigen presentation	ex. 2	Mammals: macaques	*Macaca*	[86]
MHC	MHC IIB (DQB)	antigen presentation	ex. 2	Mammals: primates	Primates	[27]
MHC	MHC IIB (DQB)	antigen presentation	ex. 2	Mammals: primates	Primates	[87]
MHC	MHC IIB (DQB)	antigen presentation	ex. 2	Mammals: macaques	*Macaca*	[88]
MHC	MHC IIB (DQB)	antigen presentation	ex. 2	Mammals: ruminants	within Cervinae and beetween Cervinae and Caprinae	[75]
MHC	MHC IIB (DQB)	antigen presentation	ex. 2	Mammals: bovids	Bovinae	[89]
MHC	MHC IIB (DQB)	antigen presentation	ex. 2	Mammals: dolphins	Delphinidae	[90]
MHC	MHC IIB (DQB)	antigen presentation	ex. 2	Mammals: cetaceans, primates, even-toed ungulates	Cetacea, Primates, Artiodactyla	[91]
MHC	MHC IIB (DQB)	antigen presentation	ex. 2	Mammals: hyeanas	Hyaenidae	[70]
MHC	MHC IIB (DQB)	antigen presentation	ex. 2	Mammals: canids	Canidae	[31]
MHC	MHC IIB (DQB)	antigen presentation	ex. 2	Mammals: bears	Ursiadae	[9]
MHC	MHC IIB (DRB like)	antigen presentation	(ex. 1, 2, 3)	Birds: penguins	*Spheniscus*	[92]
MHC	MHC IIB (DRB like)	antigen presentation	ex. 2	Birds: penguins	*Spheniscus*	[93]

Gene group	Gene name	Gene function	Gene subregion	Taxon	Taxon (scientific)	Ref.
MHC	MHC IIB (DAB)	antigen presentation	ex. 2	Mammals: mouse opossums	*Gracilinanus microtarsus* and *Marmosops incanus*	[94]
MHC	MHC IIB (DAB)	antigen presentation	ex. 2	Birds: herons	Ardeidae	[95]
MHC	MHC IIB (DAB)	antigen presentation	ex. 2	Birds: owls	Strygidae	[96]
MHC	MHC IIB (DAB)	antigen presentation	(ex. 2, 3)	Fish: cyprinids	Cyprinae, Leucisnae, Gobionianae, Tincinae, Cyprininae	[97]
MHC	MHC IIB (DAB)	antigen presentation	ex. 2	Fish: salmonids	*Salmo*	[47]
MHC	MHC IIB (DAB)	antigen presentation	ex. 2	Fish: danio	*Danio*	[48]
MHC	MHC IIB (DAB)	antigen presentation	ex. 2	Fish: cyprinids	*Chondrostoma*	[98]
MHC	MHC IIB (undif.)	antigen presentation	ex. 2	Mammals: mice	*Mus*	[49]
MHC	MHC IIB (undif.)	antigen presentation	ex. 2	Mammals: felids	Felidae	[99]
MHC	MHC IIB (undif.)	antigen presentation	ex. 2	Birds: thin-billed prion and blue petrel	between *Pachyptila belcheri* and *Halobaena caerulea*	[100]
MHC	MHC IIB (undif.)	antigen presentation	ex. 2	Birds: falcons and volture	within *Falco* and *Gyps*	[101]
MHC	MHC IIB (undif.)	antigen presentation	ex. 2	Birds: thrush nightingale and bluethroat	*Luscinia luscinia* and *Luscinia svecica*	[102]
MHC	MHC IIB (undif.)	antigen presentation	ex. 2	Birds: songbirds	*Nesospiza* and *Rowettia*	[103]
MHC	MHC IIB (undif.)	antigen presentation	ex. 2	Birds: drepanids	Drepanidinae	[104]
MHC	MHC IIB (undif.)	antigen presentation	ex. 2	Birds: Darwin's Finches and their close relatives	several tribes	[105]
MHC	MHC IIB (undif.)	antigen presentation	ex. 3	Reptiles: crocodiles	Crocodilia	[50]
MHC	MHC IIB (undif.)	antigen presentation	ex. 2	Amphibian: frogs	Ranidae	[106]
MHC	MHC IIB (undif.)	antigen presentation	ex. 2	Amphibian: frogs	*Ranna*	[107]
MHC	MHC IIB (undif.)	antigen presentation	ex. 2	Fish: sticklebacks	*Gasterosteus aculeatus* and *Pungitius pungitius*	[108]
MHC	MHC IIB (undif.)	antigen presentation	ex. 2	Fish: cyprinids	Leuciscinae	[109]
MHC	MHC IIB (undif.)	antigen presentation	ex. 2	Fish: salmonids	Salmonidae	[110]
MHC	MHC IIB (undif.)	antigen presentation	ex. 2	Fish: salmonids	Salmonidae	[111]
MHC	MHC IIB (undif.)	antigen presentation	ex. 2	Fish: white fish with other salmonids	*Coregonus* with other geni wihin salmonids	[22]
Non-MHC Ig	PSMB8	proteolytic cleveage within immunoproteasome	(ex. 2, 3)	Fish: bone fish	between and within Salmoniformes and Cypriniformes	[112]
Non-MHC Ig	PSMB8	proteolytic cleveage within immunoproteasome	(ex. 2, 3)	Fish: ricefishes	*Oryzias*	[113]
Non-MHC Ig	PSMB8	proteolytic cleveage within immunoproteasome	CDS	Amhibians: frogs	*Xenopus*	[114]
Non-MHC Ig	PSMB8	proteolytic cleveage within immunoproteasome	CDS	Fish: ray-finned fish	Osteoglossiformes and Osmeriformes	[115]
Non-MHC Ig	IgVH	antigen binding at VH domain of antibody	gene fragment	Mammals: hares and rabbits	*Lepus* and *Oryctolagus*	[116]
Non-MHC Ig	IgVH	antigen binding at VH domain of antibody	gene fragment	Mammals: hares and rabbits	*Lepus* and *Oryctolagus*	[117]

Gene group	Gene name	Gene function	Gene subregion	Taxon	Taxon (scientific)	Ref.
Non-MHC Ig	C Alfa gene	constant domain of IgA - hinge region	ex. 2	Mammals: macaques	*Macaca*	[118]
Non-MHC Ig	C Alfa gene	constant domain of IgA - hinge region	ex. 2	Mammals: new world monkeys, old world monkeyes	Catyrnii and Platyrrhini	[119]
HDP	AvBD12	disruption of bacterial cytoplasmic membrane	ex. 3	Birds: tits	*Parus major* and *Cyanistes caeruleus*	[120]
2-5A synthetase	OAS1b	activation of RNAse followed by dsRNA degradation	ex. and int.	Mammals: mice	*Mus*	[121]
2-5A synthetase	OAS1	activation of RNAse followed by dsRNA degradation	CDS	Mammals: hominoids	Hominoidea	[122]
TRIM	TRIM5alfa	restriction viral factor - capsid interfering protein	CDS	Mammals: macaques	*Macaca*	[123]
TRIM	TRIM5alfa	restriction viral factor - capsid interfering protein	int. 1	Mammals: human and chimpanze	*Pan* and *Homo*	[124]

References

1. Cao Y, Li A, Li L, Yan X, Fa Y, Zeng L, Fan J, Liu B, Sun Z: **Identification of 32 major histocompatibility complex class I alleles in African green monkeys**. *Tissue Antigens* 2014, **84**(3):304-307.
2. Satta Y, Ohuigin C, Takahata N, Klein J: **THE SYNONYMOUS SUBSTITUTION RATE OF THE MAJOR HISTOCOMPATIBILITY COMPLEX LOCI IN PRIMATES**. *Proceedings of the National Academy of Sciences of the United States of America* 1993, **90**(16):7480-7484.
3. Mayer WE, Jonker M, Klein D, Ivanyi P, Vanseventer G, Klein J: **NUCLEOTIDE-SEQUENCES OF CHIMPANZEE MHC CLASS-I ALLELES - EVIDENCE FOR TRANS-SPECIES MODE OF EVOLUTION**. *Embo Journal* 1988, **7**(9):2765-2774.
4. Suarez CF, Cardenas PP, Llanos-Ballestas EJ, Martinez P, Obregon M, Patarroyo ME, Patarroyo MA: **alpha(1) and alpha(2) domains of Aotus MHC Class I and Catarrhini MHC class Ia share similar characteristics**. *Tissue Antigens* 2003, **61**(5):362-373.
5. Babik W, Kawalko A, Wojcik JM, Radwan J: **Low Major Histocompatibility Complex Class I (MHC I) Variation in the European Bison (Bison bonasus)**. *Journal of Heredity* 2012, **103**(3):349-359.
6. Xu SX, Ren WH, Li SZ, Wei FW, Zhou KY, Yang G: **Sequence Polymorphism and Evolution of Three Cetacean MHC Genes**. *Journal of Molecular Evolution* 2009, **69**(3):260-275.
7. Sin YW, Dugdale HL, Newman C, Macdonald DW, Burke T: **Evolution of MHC class I genes in the European badger (Meles meles)**. *Ecology and Evolution* 2012, **2**(7):1644-1662.
8. Castro-Prieto A, Wachter B, Melzheimer J, Thalwitzer S, Sommer S: **Diversity and Evolutionary Patterns of Immune Genes in Free-Ranging Namibian Leopards (Panthera pardus pardus)**. *Journal of Heredity* 2011, **102**(6):653-665.
9. Kuduk K, Babik W, Bojarska K, Sliwinska EB, Kindberg J, Taberlet P, Swenson JE, Radwan J: **Evolution of major histocompatibility complex class I and class II genes in the brown bear**. *Bmc Evolutionary Biology* 2012, **12**:14.

10. Karlsson M, Westerdahl H: **Characteristics of MHC Class I Genes in House Sparrows Passer domesticus as Revealed by Long cDNA Transcripts and Amplicon Sequencing**. *Journal of Molecular Evolution* 2013, **77**(1-2):8-21.
11. Richardson DS, Westerdahl H: **MHC diversity in two Acrocephalus species: the outbred Great reed warbler and the inbred Seychelles warbler**. *Mol Ecol* 2003, **12**(12):3523-3529.
12. Yuan XY, Zeng XM, Guo XG: **MHC Class I Exon 4 in the Multiocellated Racerunners (Eremias multiocellata): Polymorphism, Duplication and Selection**. *Asian Herpetological Research* 2014, **5**(2):91-103.
13. Glaberman S, Caccone A: **Species-specific evolution of class I MHC genes in iguanas (Order : Squamata; subfamily : Iguaninae)**. *Immunogenetics* 2008, **60**(7):371-382.
14. Stiebens VA, Merino SE, Chain FJJ, Eizaguirre C: **Evolution of MHC class I genes in the endangered loggerhead sea turtle (Caretta caretta) revealed by 454 amplicon sequencing**. *Bmc Evolutionary Biology* 2013, **13**:11.
15. Zhao MA, Wang YZ, Shen H, Li CL, Chen C, Luo ZH, Wu H: **Evolution by selection, recombination, and gene duplication in MHC class I genes of two Rhacophoridae species**. *Bmc Evolutionary Biology* 2013, **13**:13.
16. Kiemnec-Tyburczy KM, Richmond JQ, Savage AE, Lips KR, Zamudio KR: **Genetic diversity of MHC class I loci in six non-model frogs is shaped by positive selection and gene duplication**. *Heredity* 2012, **109**(3):146-155.
17. Bos DH, Waldman B: **Evolution by recombination and transspecies polymorphism in the MHC class I gene of Xenopus laevis**. *Molecular Biology and Evolution* 2006, **23**(1):137-143.
18. Wang DQ, Zhong L, Wei QW, Gan XN, He SP: **Evolution of MHC class I genes in two ancient fish, paddlefish (Polyodon spathula) and Chinese sturgeon (Acipenser sinensis)**. *Febs Letters* 2010, **584**(15):3331-3339.
19. Liu JX, Ely B: **Evolution of an MHC class Ia gene fragment in four North American Morone species**. *Journal of Fish Biology* 2010, **76**(8):1984-1994.
20. O'Farrell B, Benzie JAH, McGinnity P, de Eyto E, Dillane E, Coughlan J, Cross TF: **Selection and Phylogenetics of Salmonid MHC Class I: Wild Brown Trout (Salmo trutta) Differ from a Non-Native Introduced Strain**. *Plos One* 2013, **8**(5):14.
21. Kiryu I, Dijkstra JM, Sarder RI, Fujiwara A, Yoshiura Y, Ototake M: **New MHC class Ia domain lineages in rainbow trout (Oncorhynchus mykiss) which are shared with other fish species**. *Fish & Shellfish Immunology* 2005, **18**(3):243-254.
22. Binz T, Largiader C, Muller R, Wedekind C: **Sequence diversity of Mhc genes in lake whitefish**. *Journal of Fish Biology* 2001, **58**(2):359-373.
23. Garrigan D, Hedrick PN: **Class I MHC polymorphism and evolution in endangered California Chinook and other Pacific salmon**. *Immunogenetics* 2001, **53**(6):483-489.
24. Alvarez M, MartinezLaso J, Varela P, DiazCampos N, GomezCasado E, VargasAlarcon G, GarciaTorre C, ArnaizVillena A: **High polymorphism of Mhc-E locus in non-human primates: Alleles with identical exon 2 and 3 are found in two different species**. *Tissue Antigens* 1997, **49**(2):160-167.
25. Castro MJ, Morales P, FernandezSoria V, Suerez B, Recio MJ, Alvarez M, MartinVilla M, ArnaizVillena A: **Allelic diversity at the primate Mhc-G locus: Exon 3 bears stop codons in all Cercophitecinae sequences**. *Immunogenetics* 1996, **43**(6):327-336.

26. Otting N, Bontrop RE: **EVOLUTION OF THE MAJOR HISTOCOMPATIBILITY COMPLEX DPA1 LOCUS IN PRIMATES**. *Human Immunology* 1995, **42**(2):184-187.
27. Kriener K, O'HUigin C, Klein J: **Independent origin of functional MHC class II genes in humans and New World monkeys**. *Human Immunology* 2001, **62**(1):1-14.
28. Kundu S, Faulkes CG: **A tangled history: patterns of major histocompatibility complex evolution in the African mole-rats (Family : Bathyergidae)**. *Biological Journal of the Linnean Society* 2007, **91**(3):493-503.
29. Ballingall KT, Rocchi MS, McKeever DJ, Wright F: **Trans-Species Polymorphism and Selection in the MHC Class II DRA Genes of Domestic Sheep**. *Plos One* 2010, **5**(6):10.
30. Sena L, Schneider MPC, Brenig B, Honeycutt RL, Womack JE, Skow LC: **Polymorphisms in MHC-DRA and -DRB alleles of water buffalo (Bubalus bubalis) reveal different features from cattle DR alleles**. *Animal Genetics* 2003, **34**(1):1-10.
31. Arbanasic H, Huber D, Kusak J, Gomercic T, Hrenovic J, Galov A: **Extensive polymorphism and evidence of selection pressure on major histocompatibility complex DLA-DRB1, DQA1 and DQB1 class II genes in Croatian grey wolves**. *Tissue Antigens* 2013, **81**(1):19-27.
32. Kamath PL, Getz WM: **Adaptive molecular evolution of the Major Histocompatibility Complex genes, DRA and DQA, in the genus Equus**. *Bmc Evolutionary Biology* 2011, **11**:16.
33. Janova E, Matiasovic J, Vahala J, Vodicka R, Van Dyk E, Horin P: **Polymorphism and selection in the major histocompatibility complex DRA and DQA genes in the family Equidae**. *Immunogenetics* 2009, **61**(7):513-527.
34. Loisel DA, Rockman MV, Wray GA, Altmann J, Alberts SC: **Ancient polymorphism and functional variation in the primate MHC-DQA1 5 ' cis-regulatory region**. *Proceedings of the National Academy of Sciences of the United States of America* 2006, **103**(44):16331-16336.
35. de Bellocq JG, Suchentrunk F, Baird SJE, Schaschl H: **Evolutionary history of an MHC gene in two leporid species: characterisation of Mhc-DQA in the European brown hare and comparison with the European rabbit**. *Immunogenetics* 2009, **61**(2):131-144.
36. Cutrera AP, Lacey EA: **Trans-species polymorphism and evidence of selection on class II MHC loci in tuco-tucos (Rodentia : Ctenomyidae)**. *Immunogenetics* 2007, **59**(12):937-948.
37. Koutsogiannouli EA, Moutou KA, Sarafidou T, Stamatis C, Spyrou V, Mamuris Z: **Major histocompatibility complex variation at class II DQA locus in the brown hare (Lepus europaeus)**. *Molecular Ecology* 2009, **18**(22):4631-4649.
38. Surridge AK, van der Loo W, Abrantes J, Carneiro M, Hewitt GM, Esteves PJ: **Diversity and evolutionary history of the MHC DQA gene in leporids**. *Immunogenetics* 2008, **60**(9):515-525.
39. Bryja J, Galan M, Charbonnel N, Cosson JF: **Duplication, balancing selection and trans-species evolution explain the high levels of polymorphism of the DQA MHC class II gene in voles (Arvicolinae)**. *Immunogenetics* 2006, **58**(2-3):191-202.
40. Sin YW, Dugdale HL, Newman C, Macdonald DW, Burke T: **MHC class II genes in the European badger (Meles meles): characterization, patterns of variation, and transcription analysis**. *Immunogenetics* 2012, **64**(4):313-327.
41. Moutou KA, Koutsogiannouli EA, Stamatis C, Billinis C, Kalbe C, Scandura M, Mamuris Z: **Domestication does not narrow MHC diversity in Sus scrofa**. *Immunogenetics* 2013, **65**(3):195-209.

42. Zhou H, Hickford JGH, Fang Q: **Polymorphism of the DQA2 gene in goats**. *Journal of Animal Science* 2005, **83**(5):963-968.
43. Hickford JGH, Zhou H, Slow S, Fang Q: **Diversity of the ovine DQA2 gene**. *Journal of Animal Science* 2004, **82**(6):1553-1563.
44. Zhou H, Hickford JGH: **Allelic polymorphism in the ovine DQA1 gene**. *Journal of Animal Science* 2004, **82**(1):8-16.
45. Wu HL, Tong CC, Li E, Luo TL: **Insight into gene evolution within Cervidae and Bovidae through genetic variation in MHC-DQA in the black muntjac (Muntiacus crinifrons)**. *Genetics and Molecular Research* 2012, **11**(3):2888-2898.
46. Archie EA, Henry T, Maldonado JE, Moss CJ, Poole JH, Pearson VR, Murray S, Alberts SC, Fleischer RC: **Major histocompatibility complex variation and evolution at a single, expressed DQA locus in two genera of elephants**. *Immunogenetics* 2010, **62**(2):85-100.
47. Stet RJM, de Vries B, Mudde K, Hermsen T, van Heerwaarden J, Shum BP, Grimholt U: **Unique haplotypes of co-segregating major histocompatibility class II A and class II B alleles in Atlantic salmon (Salmo salar) give rise to diverse class II genotypes**. *Immunogenetics* 2002, **54**(5):320-331.
48. Graser R, OhUigin C, Vincek V, Meyer A, Klein J: **Trans-species polymorphism of class II Mhc loci in danio fishes**. *Immunogenetics* 1996, **44**(1):36-48.
49. Cizkova D, de Bellocq JG, Baird SJE, Pialek J, Bryja J: **Genetic structure and contrasting selection pattern at two major histocompatibility complex genes in wild house mouse populations**. *Heredity* 2011, **106**(5):727-740.
50. Jaratlerdsiri W, Isberg SR, Higgins DP, Ho SYW, Salomonsen J, Skjodt K, Miles LG, Gongora J: **Evolution of MHC class I in the Order Crocodylia**. *Immunogenetics* 2014, **66**(1):53-65.
51. Sano K, Shiina T, Kohara S, Yanagiya K, Hosomichi K, Shimizu S, Anzai T, Watanabe A, Ogasawara K, Torii R *et al*: **Novel cynomolgus macaque MHC-DPB1 polymorphisms in three South-East Asian populations**. *Tissue Antigens* 2006, **67**(4):297-306.
52. Slierendregt BL, Otting N, Kenter M, Bontrop RE: **ALLELIC DIVERSITY AT THE MHC-DP LOCUS IN RHESUS MACAQUES (MACACA-MULATTA)**. *Immunogenetics* 1995, **41**(1):29-37.
53. Yasukochi Y, Satta Y: **A human-specific allelic group of the MHC DRB1 gene in primates**. *Journal of Physiological Anthropology* 2014, **33**:9.
54. !!! INVALID CITATION !!!
55. Slierendregt BL, Otting N, Vanbesouw N, Jonker M, Bontrop RE: **EXPANSION AND CONTRACTION OF RHESUS MACAQUE DRB REGIONS BY DUPLICATION AND DELETION**. *Journal of Immunology* 1994, **152**(5):2298-2307.
56. Slierendregt BL, Vannoort JT, Bakas RM, Otting N, Jonker M, Bontrop RE: **EVOLUTIONARY STABILITY OF TRANSSPECIES MAJOR HISTOCOMPATIBILITY COMPLEX CLASS-II DRB-LINEAGES IN HUMANS AND RHESUS-MONKEYS**. *Human Immunology* 1992, **35**(1):29-39.
57. Gaur LK, Nepom GT, Snyder KE, Anderson J, Pandarpurkar M, Yadock W, Heise ER: **MHC-DRB allelic sequences incorporate distinct intragenic trans-specific segments**. *Tissue Antigens* 1997, **49**(4):342-355.
58. Kriener K, O'HUigin C, Tichy H, Klein J: **Convergent evolution of major histocompatibility complex molecules in humans and New World monkeys**. *Immunogenetics* 2000, **51**(3):169-178.
59. Corell A, Morales P, Varela P, Pazartal E, Martinvilla JM, Martinezlaso J, Arnaizvillena A: **ALLELIC DIVERSITY AT THE PRIMATE MAJOR HISTOCOMPATIBILITY COMPLEX DRB6 LOCUS**. *Immunogenetics* 1992, **36**(1):33-38.

60. Kupfermann H, Mayer WE, Ohuigin C, Klein D, Klein J: **SHARED POLYMORPHISM BETWEEN GORILLA AND HUMAN MAJOR HISTOCOMPATIBILITY COMPLEX DRB LOCI**. *Human Immunology* 1992, **34**(4):267-278.
61. Brandle U, Ono H, Vincek V, Klein D, Golubic M, Grahovac B, Klein J: **TRANSSPECIES EVOLUTION OF MHC-DRB HAPLOTYPE POLYMORPHISM IN PRIMATES - ORGANIZATION OF DRB GENES IN THE CHIMPANZEE**. *Immunogenetics* 1992, **36**(1):39-48.
62. Geluk A, Elferink DG, Slierendregt BL, Vanmeijgaarden KE, Devries RRP, Ottenhoff THM, Bontrop RE: **EVOLUTIONARY CONSERVATION OF MAJOR HISTOCOMPATIBILITY COMPLEX-DR/PEPTIDE/T-CELL INTERACTIONS IN PRIMATES**. *Journal of Experimental Medicine* 1993, **177**(4):979-987.
63. Mayer WE, Ohuigin C, Zaleskarutczynska Z, Klein J: **TRANSSPECIES ORIGIN OF MHC-DRB POLYMORPHISM IN THE CHIMPANZEE**. *Immunogenetics* 1992, **37**(1):12-23.
64. Go Y, Satta Y, Kawamoto Y, Rakotoarisoa G, Randrianjafy A, Koyama N, Hirai H: **Mhc-DRB genes evolution in lemurs**. *Immunogenetics* 2002, **54**(6):403-417.
65. Musolf K, Meyer-Lucht Y, Sommer S: **Evolution of MHC-DRB class II polymorphism in the genus Apodemus and a comparison of DRB sequences within the family Muridae (Mammalia : Rodentia)**. *Immunogenetics* 2004, **56**(6):420-426.
66. de Bellocq JG, Charbonnel N, Morand S: **Coevolutionary relationship between helminth diversity and MHC class II polymorphism in rodents**. *Journal of Evolutionary Biology* 2008, **21**(4):1144-1150.
67. Winternitz JC, Wares JP: **Duplication and population dynamics shape historic patterns of selection and genetic variation at the major histocompatibility complex in rodents**. *Ecology and Evolution* 2013, **3**(6):1552-1568.
68. Goda N, Mano T, Kosintsev P, Vorobiev A, Masuda R: **Allelic diversity of the MHC class II DRB genes in brown bears (Ursus arctos) and a comparison of DRB sequences within the family Ursidae**. *Tissue Antigens* 2010, **76**(5):404-410.
69. Wan QH, Zhu L, Wu H, Fang SG: **Major histocompatibility complex class II variation in the giant panda (Ailuropoda melanoleuca)**. *Molecular Ecology* 2006, **15**(9):2441-2450.
70. Califf KJ, Ratzloff EK, Wagner AP, Holekamp KE, Williams BL: **Forces shaping major histocompatibility complex evolution in two hyena species**. *Journal of Mammalogy* 2013, **94**(2):282-294.
71. Hedrick PW, Lee RN, Parker KM: **Major histocompatibility complex (MHC) variation in the endangered Mexican wolf and related canids**. *Heredity* 2000, **85**(6):617-624.
72. Becker L, Nieberg C, Jahreis K, Peters E: **MHC class II variation in the endangered European mink Mustela lutreola (L. 1761)-consequences for species conservation**. *Immunogenetics* 2009, **61**(4):281-288.
73. O'Brien SJ, Yuhki N: **Comparative genome organization of the major histocompatibility complex: lessons from the Felidae**. *Immunological Reviews* 1999, **167**:133-144.
74. Schaschl H, Wandeler P, Suchentrunk F, Obexer-Ruff G, Goodman SJ: **Selection and recombination drive the evolution of MHC class II DRB diversity in ungulates**. *Heredity* 2006, **97**(6):427-437.
75. Liu HY, Xue F, Wan QH, Ge YF: **MHC Class II beta Genes in the Endangered Hainan Eld's Deer (Cervus eldi hainanus)**. *Journal of Heredity* 2013, **104**(6):874-880.

76. Mikko S, Spencer M, Morris B, Stabile S, Basu T, Stormont C, Andersson L: **A comparative analysis of Mhc DRB3 polymorphism in the American bison (Bison bison)**. *Journal of Heredity* 1997, **88**(6):499-503.
77. Worley K, Carey J, Veitch A, Coltman DW: **Detecting the signature of selection on immune genes in highly structured populations of wild sheep (Ovis dalli)**. *Molecular Ecology* 2006, **15**(3):623-637.
78. Mona S, Crestanello B, Bankhead-Dronnet S, Pecchioli E, Ingrosso S, D'Amelio S, Rossi L, Meneguz PG, Bertorelle G: **Disentangling the effects of recombination, selection, and demography on the genetic variation at a major histocompatibility complex class II gene in the alpine chamois**. *Molecular Ecology* 2008, **17**(18):4053-4067.
79. Mikko S, Roed K, Schmutz S, Andersson L: **Monomorphism and polymorphism at Mhc DRB loci in domestic and wild ruminants**. *Immunological Reviews* 1999, **167**:169-178.
80. Van Den Bussche RA, Hoofer SR, Lochmiller RL: **Characterization of Mhc-DRB allelic diversity in white-tailed deer (Odocoileus virginianus) provides insight into Mhc-DRB allelic evolution within Cervidae**. *Immunogenetics* 1999, **49**(5):429-437.
81. Van der Walt JM, Nel LH, Hoelzel AR: **Characterization of major histocompatibility complex DRB diversity in the endemic South African antelope Damaliscus pygargus: a comparison in two subspecies with different demographic histories**. *Molecular Ecology* 2001, **10**(7):1679-1688.
82. Li B, Xu YC, Ma JZ: **Allelic characterization of the second DRB locus of major histocompatibility complex class II in Ussuri sika deer (Cervus nippon hortulorum): highlighting the trans-species evolution of DRB alleles within Cervidae**. *Animal Cells and Systems* 2013, **17**(4):269-276.
83. Schad J, Voigt CC, Greiner S, Dechmann DKN, Sommer S: **Independent evolution of functional MHC class II DRB genes in New World bat species**. *Immunogenetics* 2012, **64**(7):535-547.
84. Walsh HE, Friesen VL: **A comparison of intraspecific patterns of DNA sequence variation in mitochondrial DNA, alpha-enolase, and MHC class II B loci in auklets (Charadriiformes : Alcidae)**. *Journal of Molecular Evolution* 2003, **57**(6):681-693.
85. Otting N, Kenter M, Vanweeren P, Jonker M, Bontrop RE: **MHC-DQB REPERTOIRE VARIATION IN HOMINOID AND OLD-WORLD PRIMATE SPECIES**. *Journal of Immunology* 1992, **149**(2):461-470.
86. Yao YF, Dai QX, Li J, Ni QY, Zhang MW, Xu HL: **Genetic diversity and differentiation of the rhesus macaque (Macaca mulatta) population in western Sichuan, China, based on the second exon of the major histocompatibility complex class II DQB (MhcMamu-DQB1) alleles**. *Bmc Evolutionary Biology* 2014, **14**:13.
87. Otting N, de Groot NG, Doxiadis GGM, Bontrop RE: **Extensive Mhc-DQB variation in humans and non-human primate species**. *Immunogenetics* 2002, **54**(4):230-239.
88. Yao YF, Zhao JJ, Dai QX, Li JY, Zhou L, Wang YT, Ni QY, Zhang MW, Xu HL: **Identification and characterization of the major histocompatibility complex class II DQB (MhcMath-DQB1) alleles in Tibetan macaques (Macaca thibetana)**. *Tissue Antigens* 2013, **82**(2):113-121.
89. Sena L, Schneider MPC, Brenig BB, Honeycutt RL, Honeycutt DA, Womack JE, Skow LC: **Polymorphism and gene organization of water buffalo MHC-DQB genes show homology to the BoLA DQB region**. *Animal Genetics* 2011, **42**(4):378-385.

90. Arbanasic H, Duras M, Podnar M, Gomercic T, Curkovic S, Galov A: **Major histocompatibility complex class II variation in bottlenose dolphin from Adriatic Sea: inferences about the extent of balancing selection**. *Marine Biology* 2014, **161**(10):2407-2422.
91. Villanueva-Noriega MJ, Baker CS, Medrano-Gonzalez L: **Evolution of the MHC-DQB exon 2 in marine and terrestrial mammals**. *Immunogenetics* 2013, **65**(1):47-61.
92. Kikkawa EF, Tsuda TT, Sumiyama D, Naruse TK, Fukuda M, Kurita M, Wilson RP, LeMaho Y, Miller GD, Tsuda M *et al*: **Trans-species polymorphism of the Mhc class II DRB-like gene in banded penguins (genus Spheniscus)**. *Immunogenetics* 2009, **61**(5):341-352.
93. Bollmer JL, Vargas FH, Parker PG: **Low MHC variation in the endangered Galapagos penguin (Spheniscus mendiculus)**. *Immunogenetics* 2007, **59**(7):593-602.
94. Meyer-Lucht Y, Otten C, Puttker T, Sommer S: **Selection, diversity and evolutionary patterns of the MHC class II DAB in free-ranging Neotropical marsupials**. *Bmc Genetics* 2008, **9**:14.
95. Li L, Zhou XP, Chen XL: **Characterization and Evolution of MHC Class II B Genes in Ardeid Birds**. *Journal of Molecular Evolution* 2011, **72**(5-6):474-483.
96. Burri R, Hirzel HN, Salamin N, Roulin A, Fumagalli L: **Evolutionary patterns of MHC class IIB in owls and their implications for the understanding of avian MHC evolution**. *Molecular Biology and Evolution* 2008, **25**(6):1180-1191.
97. Ottova E, Simkova A, Martin JF, de Bellocq JG, Gelnar M, Allienne JF, Morand S: **Evolution and trans-species polymorphism of MHC class II beta genes in cyprinid fish**. *Fish & Shellfish Immunology* 2005, **18**(3):199-222.
98. Simkova A, Civanova K, Gettova L, Gilles A: **Genomic Porosity between Invasive Chondrostoma nasus and Endangered Endemic Parachondrostoma toxostoma (Cyprinidae): The Evolution of MHC IIB Genes**. *Plos One* 2013, **8**(6):16.
99. Wei K, Zhang ZH, Wang XF, Zhang WP, Xu X, Shen FJ, Yue BS: **Lineage pattern, trans-species polymorphism, and selection pressure among the major lineages of feline Mhc-DRB peptide-binding region**. *Immunogenetics* 2010, **62**(5):307-317.
100. Strandh M, Lannefors M, Bonadonna F, Westerdahl H: **Characterization of MHC class I and II genes in a subantarctic seabird, the blue petrel, Halobaena caerulea (Procellariiformes)**. *Immunogenetics* 2011, **63**(10):653-666.
101. Alcaide M, Edwards SV, Negro JJ: **Characterization, polymorphism, and evolution of MHC class IIB genes in birds of prey**. *Journal of Molecular Evolution* 2007, **65**(5):541-554.
102. Anmarkrud JA, Johnsen A, Bachmann L, Lifjeld JT: **Ancestral polymorphism in exon 2 of bluethroat (Luscinia svecica) MHC class II B genes**. *Journal of Evolutionary Biology* 2010, **23**(6):1206-1217.
103. van Rensburg AJ, Bloomer P, Ryan PG, Hansson B: **Ancestral polymorphism at the major histocompatibility complex (MHCII ss) in the Nesospiza bunting species complex and its sister species (Rowettia goughensis)**. *Bmc Evolutionary Biology* 2012, **12**.
104. Jarvi SI, Tarr CL, McIntosh CE, Atkinson CT, Fleischer RC: **Natural selection of the major histocompatibility complex (Mhc) in Hawaiian honeycreepers (Drepanidinae)**. *Mol Ecol* 2004, **13**(8):2157-2168.
105. Sato A, Tichy H, Grant PR, Grant BR, Sato T, O'HUigin C: **Spectrum of MHC Class II Variability in Darwin's Finches and Their Close Relatives**. *Molecular Biology and Evolution* 2011, **28**(6):1943-1956.

106. Shu YL, Hong P, Yang YW, Wu HL: **An Endemic Frog Harbors Multiple Expression Loci With Different Patterns of Variation in the MHC Class II B Gene**. *Journal of Experimental Zoology Part B-Molecular and Developmental Evolution* 2013, **320**(8):501-510.
107. Kiemnec-Tyburczy KM, Richmond JQ, Savage AE, Zamudio KR: **Selection, trans-species polymorphism, and locus identification of major histocompatibility complex class II beta alleles of New World ranid frogs**. *Immunogenetics* 2010, **62**(11-12):741-751.
108. Lenz TL, Eizaguirre C, Kalbe M, Milinski M: **EVALUATING PATTERNS OF CONVERGENT EVOLUTION AND TRANS-SPECIES POLYMORPHISM AT MHC IMMUNOGENES IN TWO SYMPATRIC STICKLEBACK SPECIES**. *Evolution* 2013, **67**(8):2400-2412.
109. Seifertova M, Simkova A: **Structure, diversity and evolutionary patterns of expressed MHC class IIB genes in chub (Squalius cephalus), a cyprinid fish species from Europe**. *Immunogenetics* 2011, **63**(3):167-181.
110. Peters MB, Turner TF: **Genetic variation of the major histocompatibility complex (MHC class II beta gene) in the threatened Gila trout, Oncorhynchus gilae gilae**. *Conservation Genetics* 2008, **9**(2):257-270.
111. Aguilar A, Garza JC: **Patterns of historical balancing selection on the salmonid major histocompatibility complex class II beta gene**. *Journal of Molecular Evolution* 2007, **65**(1):34-43.
112. Tsukamoto K, Miura F, Fujito NT, Yoshizaki G, Nonaka M: **Long-Lived Dichotomous Lineages of the Proteasome Subunit Beta Type 8 (PSMB8) Gene Surviving More than 500 Million Years as Alleles or Paralogs**. *Molecular Biology and Evolution* 2012, **29**(10):3071-3079.
113. Miura F, Tsukamoto K, Mehta RB, Naruse K, Magtoon W, Nonaka M: **Transspecies dimorphic allelic lineages of the proteasome subunit beta-type 8 gene (PSMB8) in the teleost genus Oryzias**. *Proceedings of the National Academy of Sciences of the United States of America* 2010, **107**(50):21599-21604.
114. Nonaka M, Yamada-Namikawa C, Flajnik MF, Du Pasquier L: **Trans-species polymorphism of the major histocompatibility complex-encoded proteasome subunit LMP7 in an amphibian genus, Xenopus**. *Immunogenetics* 2000, **51**(3):186-192.
115. Noro M, Nonaka M: **Evolution of dimorphisms of the proteasome subunit beta type 8 gene (PSMB8) in basal ray-finned fish**. *Immunogenetics* 2014, **66**(5):325-334.
116. Esteves PJ, Lanning D, Ferrand N, Knight KL, Zhai SK, van der Loo W: **The evolution of the immunoglobulin heavy chain variable region (IgV(H)) in Leporids: an unusual case of transspecies polymorphism**. *Immunogenetics* 2005, **57**(11):874-882.
117. Su C, Nei M: **Fifty-million-year-old polymorphism at an immunoglobulin variable region gene locus in the rabbit evolutionary lineage**. *Proceedings of the National Academy of Sciences of the United States of America* 1999, **96**(17):9710-9715.
118. Sumiyama K, Kawamura S, Takenaka O, Ueda S: **A high sequence variety in the immunoglobulin C-alpha hinge region among old world monkeys**. *Anthropological Science* 1998, **106**(1):31-39.
119. Sumiyama K, Saitou N, Ueda S: **Adaptive evolution of the IgA hinge region in primates**. *Molecular Biology and Evolution* 2002, **19**(7):1093-1099.
120. Hellgren O, Sheldon BC: **Locus-specific protocol for nine different innate immune genes (antimicrobial peptides: beta-defensins) across passerine bird species reveals within-species coding variation and a case of trans-species polymorphisms**. *Molecular Ecology Resources* 2011, **11**(4):686-692.

121. Ferguson W, Dvora S, Gallo J, Orth A, Boissinot S: **Long-term balancing selection at the West Nile virus resistance gene, Oas1b, maintains transspecific polymorphisms in the house mouse**. *Mol Biol Evol* 2008, **25**(8):1609-1618.
122. Ferguson W, Dvora S, Fikes RW, Stone AC, Boissinot S: **Long-Term Balancing Selection at the Antiviral Gene OAS1 in Central African Chimpanzees**. *Molecular Biology and Evolution* 2012, **29**(4):1093-1103.
123. Newman RM, Hall L, Connole M, Chen GL, Sato S, Yuste E, Diehl W, Hunter E, Kaur A, Miller GM *et al*: **Balancing selection and the evolution of functional polymorphism in Old World monkey TRIM5 alpha**. *Proceedings of the National Academy of Sciences of the United States of America* 2006, **103**(50):19134-19139.
124. Cagliani R, Fumagalli M, Biasin M, Piacentini L, Riva S, Pozzoli U, Bonaglia MC, Bresolin N, Clerici M, Sironi M: **Long-term balancing selection maintains trans-specific polymorphisms in the human TRIM5 gene**. *Human Genetics* 2010, **128**(6):577-588.
